# Prognostic relevance of telomere length and telomerase reverse transcriptase variant (rs2242652) on the multiple myeloma patients

**DOI:** 10.1002/jcla.23133

**Published:** 2019-12-08

**Authors:** Salah Aref, Alshaimaa Al Saeed, Nadia El Menshawy, Doaa Abdalla, Mohamed El Ashery

**Affiliations:** ^1^ Hematology Unit Mansoura University Oncology Center Mansoura University Mansoura Egypt; ^2^ Clinical Oncology Department Mansoura University Mansoura Egypt

**Keywords:** multiple myeloma, relative telomere length, rs2242652, TERT

## Abstract

**Background:**

The search for enhancement of multiple myeloma prognostic tools is an area of current research. This study aimed to assess the clinicopathological impact of telomere length and telomerase reverse transcriptase (TERT) polymorphic variant, rs2242652, on multiple myeloma (MM) patients.

**Methods:**

Fifty MM patients and 50 healthy controls were included. Relative telomere length (RTL) and rs2242652 genotype polymorphic variants of TERT were analyzed using real‐time polymerase chain reaction (PCR). The MM patients' group was categorized into stage I (n = 16); stage II (n = 12), and stage III (n = 22).

**Results:**

The median telomere length was significantly longer in MM patients' group (0.78) as compared to controls (0.43) (*P* = .001). Multivariate regression analysis revealed that MM patients with RTL < 0.5 had significant poor response for induction remission therapy with odds ratio 26.45. On the other hand, TERT genotyping analysis of rs2242652 revealed insignificant difference between cases and controls (*P* = .234), regarding to induction remission response. Survival analysis using Kaplan‐Meier curve revealed that patients with shorter telomere length and those with TERT genotype GA had shorter overall survival.

**Conclusion:**

Telomere length and TERT rs2242652 genotype polymorphism could be used for refining risk stratification of MM patients.

## 1 INTRODUCTION

Multiple myeloma (MM) is an age‐dependent monoclonal tumor of bone marrow plasma cells. MM accounts for 1% of all malignancies and 10% of all hematologic malignancies.^1^ The revised International Myeloma Working Group (IMWG) criteria allow, in addition to the classic CRAB features (increased calcium level, renal dysfunction, anemia, and destructive bone lesions), myeloma defining events (60% or greater clonal plasma cells on bone marrow examination, serum involved/uninvolved free light chain ratio ≥100, provided the absolute level of the involved light chain is at least 100 mg/L, and more than one focal lesion on MRI that is at least 5 mm or greater in size). The presence of at least one of these markers is considered sufficient for a diagnosis of multiple myeloma, regardless of the presence or absence of symptoms or CRAB features.^2^

Telomeres are non‐coding repetitive nucleotide sequences at the ends of all chromosomes, protecting the end of the chromosome from deterioration or from fusion with neighboring chromosomes. For vertebrates, the sequence of nucleotides in telomeres is TTAGGG, with the complementary DNA strand being AATCCC.^3^ Telomere length (TL) is maximum at birth and decreases progressively with advancing age and thus is considered as a biomarker of chronological aging.^4^ Telomere elongation is a common molecular feature of advanced malignancies.^5^, ^6^

Telomerase is a ribonucleoprotein consisting of two main components, telomere RNA component (TERC) and telomere reverse transcriptase (TERT), which act as a reverse transcriptase in the elongation of telomere length.^7^ Telomerase activity has been found in myeloma cells of 90% of the newly diagnosed and relapsed patients, while in only 13% of patients in remission.^8^ It is reported that genetic variations in TERT and TERC are involved in the many types of cancers,^9^ such as lung cancer,^10^, ^11^ breast cancer,^12^ colon cancer,^13^ and melanoma.^14^

The variant of TERT gene (rs2242652) which located on 5p15, intron 4 of TERT (encoding telomerase reverse transcriptase), seems to be associated with cancer risk as this SNP has been associated with multiple cancers, including breast, ovarian cancer,^15^ and prostate.^16^

The aim of the present study was to assess the relative telomere length and TERT gene polymorphism and their impact on survival and response to therapy in a cohort of Egyptian myeloma patients.

## 2 SUBJECTS AND METHODS

This study was carried out on 50 multiple myeloma cases (27 males and 23 females; age range 41‐78 with mean age 56.84) attending Mansoura University Oncology Center before the start of the therapy. Fifty healthy subjects (25 males and 25 females; age range 43‐80 with mean age 60.24) were included as a control group. The selected cases were diagnosed according to the International Myeloma Working Group (IMWG) criteria. The patients and controls have written informed consent. The study was approved by local ethical committee.

The studied cases were categorized into three groups according to the response to treatment with bortezomib plus melphalan‐prednisone (VMP). First group: complete response (CR) with no serum M protein by immunofixation, plasma cells <5% in bone marrow aspirate, no increase in the size or number of lytic bone lesions, and disappearance of soft‐tissue plasmacytomas. Second group: partial response (PR) with reduction in serum M protein by ≥50% and reduction in soft‐tissue plasmacytomas by ≥50%. Third group: no response (NR) to treatment when the reduction in serum M protein after 6 weeks of treatment is <25% and no increase in the size or number of lytic bone lesions.

The staging multiple myeloma was done according to the International Staging System (ISS): stage I (B2M <3.5 mg/L and serum albumin ≥3.5 g/dL), stage II (neither stage I nor stage III), and stage III (B2M ≥5.5 mg/L). The study was carried out between January 2015 and December 2017. The mean follow‐up period was 24 months.

### 2.1 Sampling

Two mL of EDTA peripheral blood samples were collected from patients and controls for complete blood count (CBC) and DNA extraction. Five mL of blood samples were left to clot to obtain sera to be tested for protein electrophoresis, serum immunofixation, LDH, B2M, creatinine, calcium, and albumin. Two mL of citrated blood samples were collected for ESR. Bone marrow aspirate and bone marrow biopsy specimens were collected from patients for morphologic and immunophenotypic diagnosis.

### 2.2 DNA extraction

DNA was extracted using Thermo scientific Gene JET Whole Blood Genomic DNA Purification Kit according to the protocol of manufacturer's instructions. The extracted DNA was stored frozen at −20°C. The DNA samples were quantified by NanoDrop instrument, and the samples were measured 17‐45 ng/μL.

### 2.3 Genotyping analysis

The detection of rs2242652 variant of TERT gene on chromosome 5 was followed the protocol of Custom TaqMan^®^ SNP Genotyping Assays manufacturing kits. The order of DNAs from cases and controls was randomized on 96‐well plate over two runs with duplication of 4 samples all over the runs for quality control purpose. PCR plates were read on DNA‐Technology DT Prime4 real‐time instrument, and software v7.6 was used to determine the genotyping.

### 2.4 Relative telomere length measurement

Leukocyte telomere length was measured using a real‐time quantitative polymerase chain reaction (RT‐PCR) technique developed by Cawthon with minor modification. Cawthon method compares signals from telomere repeat copy number (T) to a single‐copy gene copy number (S) and allows calculation of a relative T/S ratio. The primer sequences (written 5′→3′) were tel1b: 5_CGGTTTGTTTGGGTTTGGGTTTGGGTTTGGGTTTGGGTT3_; tel2b: 5_GGCTTGCCTTACCCTTACCCTTACCCTTACCCTTACCCT‐3_; 36B4u: 5′‐CAG CAA GTG GGA AGG TGT AAT CC‐3′; and 36b4d: 5′‐CCC ATT CTA TCA TCA ACG GGT ACA A‐3′**.** 18 The SYBR^®^ Green PCR Master Mix, optimized for real‐time PCR analysis, conveniently combines SYBR Green 1 Dye, AmpliTaq Gold^®^ DNA Polymerase, dNTPs with dUTP, Passive Reference 1, and optimized buffer components. A standard was prepared, by using a reference DNA sample (DNA Promega Kit) diluted to get out five serial concentrations (20‐10‐5‐2.5‐1.25 ng/μL). Telomere (T) PCRs and single‐copy gene (S) PCRs were performed in separate well plates. The order of DNAs from cases and controls was randomized on 96‐well plate over two runs with duplication of 4 samples all over the runs for quality control purpose. Each PCR well contained DNA (35 ng/aliquot), 10 μL of the SYBR^®^ Green master mix and 1 μL of forward primers, 1 μL of reverse primers specific for each plate T and S, PCR reagents and DNase‐free water to reach 20 μL/aliquot. PCRs were performed on the ViiA^™^7 system/ 96‐well block (0.2 mL), Software v1.2 (Applied Biosystems). Thermal cycling profile for both amplicons began with 95°C incubation for 10 minutes to activate the AmpliTaq Gold DNA polymerase. For telomere PCR, followed 18 cycles of 95°C for 15 seconds, 54°C for 2 minutes. For 36B4 PCR, followed 30 cycles of 95°C for 15 seconds, 58°C for 1 minutes.

### 2.5 Relative telomere length calculation

Relative T/S values were calculated according to 2^−ΔΔ Ct^
ΔCt = Ct (calibrator) − Ct (unknown sample)
ΔΔCt = ΔCt (telomere) − ΔCt (single‐copy gene).^17^, ^18^

### 2.6 Statistical analysis

Data were analyzed with SPSS version 21. The normality of data was first tested with one‐sample Kolmogorov‐Smirnov test. Association between categorical variables was tested using chi‐square test. Continuous variables were presented as mean ± SD (standard deviation) for parametric data and median for non‐parametric data. The two groups were compared with Student *t* test (parametric data) and Mann‐Whitney test (non‐parametric data). ANOVA test was used for comparison of means of more than two groups in parametric data. Spearman correlation used to correlate continuous non‐parametric data.

The results were considered significant when the probability of error *P*‐value < .05 and highly significant when the *P*‐value < .001.

## 3 RESULTS

The patient's characteristics in comparison with controls are shown in Table 1. The MM patients had significantly lower hemoglobin levels; higher serum creatinine; and lower serum albumin levels as compared to control group (*P* = .001 for all).

**Table 1:** Demographic, clinical, and laboratory data of studied groups

	Patients (n = 50)	Control (n = 50)	*P*‐value
Age (y)	56.8 ± 7.8	60.2 ± 8.9	.058
Sex			
Male n (%)	27 (54%)	25 (50%)	
Female n (%)	23(46%)	25 (50%)	.689
Hypertension, n (%)	9 (18%)	0	
Diabetes, n (%)	8(16%)	0	
Hb (g/dL)	8.48 ± 1.73	12.89 ± 1.25	.001
Ca (mg/dL)	9.24 ± 1.58	9.59 ± 0.54	.143
Creatinine (mg/dL)	1.4 (0.6‐11)	0.85 (0.5‐1.2)	.001
Albumin (g/dL)	3.36 ± 0.78	4.56 ± 0.41	.001

Note

Data presented as mean, SD, and percentage, some presented with median, range, *P* highly significant (.001).

Abbreviations: Ca, calcium; Hb, hemoglobin.

The median telomere length (TL) in MM cases was 0.78 (range: 0.21‐2.30) and in the controls was 0.43 (range: 0.20‐2.3); the difference was statistically significant (*P* = .001). The relative telomere length was classified into three subgroups to simplify the statistical result (<0.5, 0.5‐1, and >1), and then after doing chi‐square test, most of the MM patients located in the subgroup with longer telomere length (>1.0), while most of the control subjects located in the subgroup with shorter telomere length (<0.5); and the difference was statistically significant (*P* = .013) (Table 2). Moreover, the TERT genotypes (rs2242652) (AA; AG; GG) did not significantly different in MM patients as compared to controls (*P* = .234) Table 3. In addition, there is no significant association between TERT genotyping (SNP) and MM stages or induction of remission response (*P* > .05) (Table 4).

**Table 2:** Relative telomere length (RTL) in MM patients versus control one

RTL	Cases group (n = 50)	Control group (n = 50)	Test of significance *P*‐value
<0.5	17 (34%)	28 (56%)	*χ* ^2^ = 8.670 *P* = .013*
0.5‐1	12 (24%)	14 (28%)	
>1	21 (42%)	8 (16%)	
Median (Min‐Max)	0.78 (0.21‐2.30)	0.43 (0.20‐2.3)	Z = 3.192 *P* = .001*

Note

*χ* ^2^: chi‐square test, Z: Mann‐Whitney test.

* Significant *P* < .05.

**Table 3:** TERT genotyping (SNP) in the studied groups

Genotyping	Cases group (n = 50%)	Control group (n = 50%)	Test of significance *P*‐value
GG	28 (56)	35 (70)	*χ* ^2^ = 2.91
AA	15 (30)	8 (16)	*P* = .234
GA	7 (14)	7 (14)	

Abbreviations: AA, rare homozygous; GA, heterozygous; GG, common homozygous; *χ* ^2^, chi‐square test.

**Table 4:** Relation between TERT genotyping (SNP) and MM stages or induction of remission response

Variables	TERT genotype (GG)	TERT genotype (AA)	TERT genotype (GA)	Test of significance *P*‐value
Stage I (n = 16)	8 (50%)	5 (31.2%)	3 (18.8%)	*χ* ^2^ = 1.885
Stage II (n = 12)	6 (50%)	5 (41.7%)	1 (8.3%)	*P* = .757
Stage III (n = 22)	14 (63.6%)	5 (22.7%)	3 (13.6%)	
CR (n = 19)	11 (57.8%)	4 (21.1%)	4 (21.1%)	*χ* ^2^ = 3.373
PR (n = 9)	5 (55.6%)	4 (44.4%)	0 (0%)	*P* = .497
NR (n = 14)	8 (57.2%)	3 (21.4%)	3 (21.4%)	

Note

Significant *P* < .05.

Abbreviations: AA, rare homozygous; CR, complete remission; GA, heterozygous; GG, common homozygous; NR, no response; PR, partial response; SNP, single nucleotide polymorphism; *χ* ^2^, chi‐square test.

Among MM patients, younger patients are more frequently had longer telomere length; stage III MM patients frequently had shorter telomere length, while those in stage I frequently had longer telomere length. Furthermore, MM patients with shorter telomere length were non‐responder for induction therapy, while those with longer TL express more frequent complete response for induction therapy (Table 5).

**Table 5:** Relation between relative telomere length and clinical staging; induction of remission response and polymorphic TERT genotypes

Variables	RTL <0.5 (n = 17)	RTL 0.5‐1 (n = 12)	RTL >1 (n = 21)	Test of significance *P*‐value
Age/(y)	58.62 ± 5.19_a_	58.67 ± 5.39_b_	54.81 ± 10.09_ab_	*F* = 11.51 *P* = <.001**
Stage I (n = 16)	1 (6.2%)	5 (31.2%)	10 (62.5%)	*χ* ^2^ = 10.72
Stage II (n = 12)	4 (33.3%)	2 (16.7%)	6 (50.0%)	*P* = .03*
Stage III (n = 22)	12 (54.5%)	5 (22.7%)	5 (22.7%)	
CR (n = 19)	2 (13.3%)	8 (80%)	9 (53%)	*χ* ^2^ = 12.26
PR (n = 9)	5 (33.3%)	0 (0.0%)	4 (23.5%)	*P* = .016*
NR (n = 19)	8 (53.4%)	2 (20%)	4 (23.5%)	
Polymorphic TERT genotypes				
GG (n = 28)	9 (52.9%)	7 (58.3%)	12 (57.1%)	*χ* ^2^ = 0.544 *P* < .05
GA (n = 7)	3 (17.6%)	1 (8.3%)	3 (14.3%)	
AA(n = 15)	5 (29.4%)	4 (33.3%)	6 (28.6%)	

Note

A similar subscripted letter indicates significant *P* < .05.

Abbreviations: *χ* ^2^, chi‐square test; CR, complete remission; F, ANOVA test; NR, no response; PR, partial response.

* Significant

** highly significant

Using Cox regression analysis, all MM patients' parameters to predict the response of the patients for induction therapy revealed that the two most significant independent bad predictors for response to induction therapy were MM patients stages and relative telomere length <0.5 (OR 4.88 and 26.45, respectively) (Table 6).

**Table 6:** Logistic regression analysis of independent predictors of induction of remission response

Independent predictor	Univariate regression			Multivariate regression	
	*β*	*P*	COR (95% CI)	*P*	AOR (95% CI)
Hb	−0.385	.05	0.68 (0.46‐1)	–	–
Stage					
Stage l (r)	–	–	1		1
Stage ll	1.117	.209	3.06 (0.5‐17.4)	.049*	ADD AOR&CI
Stage lll	3.314	.001	27.5 (3.9‐193)		4.88 (1.5‐42.6)
RTL					
<0.05	1.990	.027*	7.31 (1.2‐42.8)	.009*	26.45 (2.3‐309)
0.5‐1	−1.269	.172	0.28 (0.05‐1.7)		ADD AOR&CI
>1 (r)	–	–	1		1
Constant	−1.01				
Model *χ* ^2^	24.3, *P* < .001				
% correctly predicted	81%				

Abbreviations: AOR, adjusted odds ratio; CI, confidence interval; COR, crude odds ratio.

* significant

**Figure 1:**
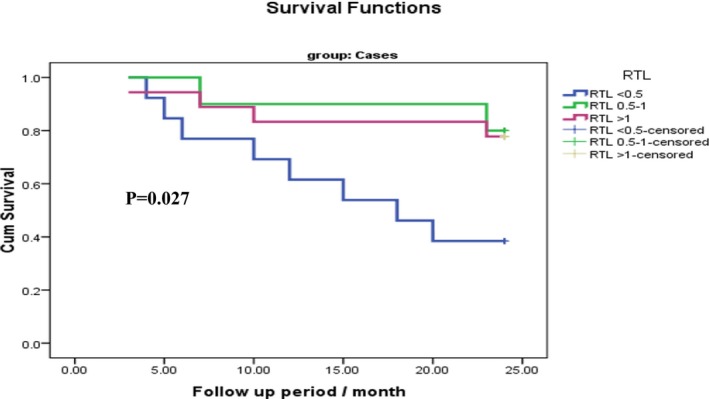
Impact of telomere length on MM patients overall survival

**Figure 2:**
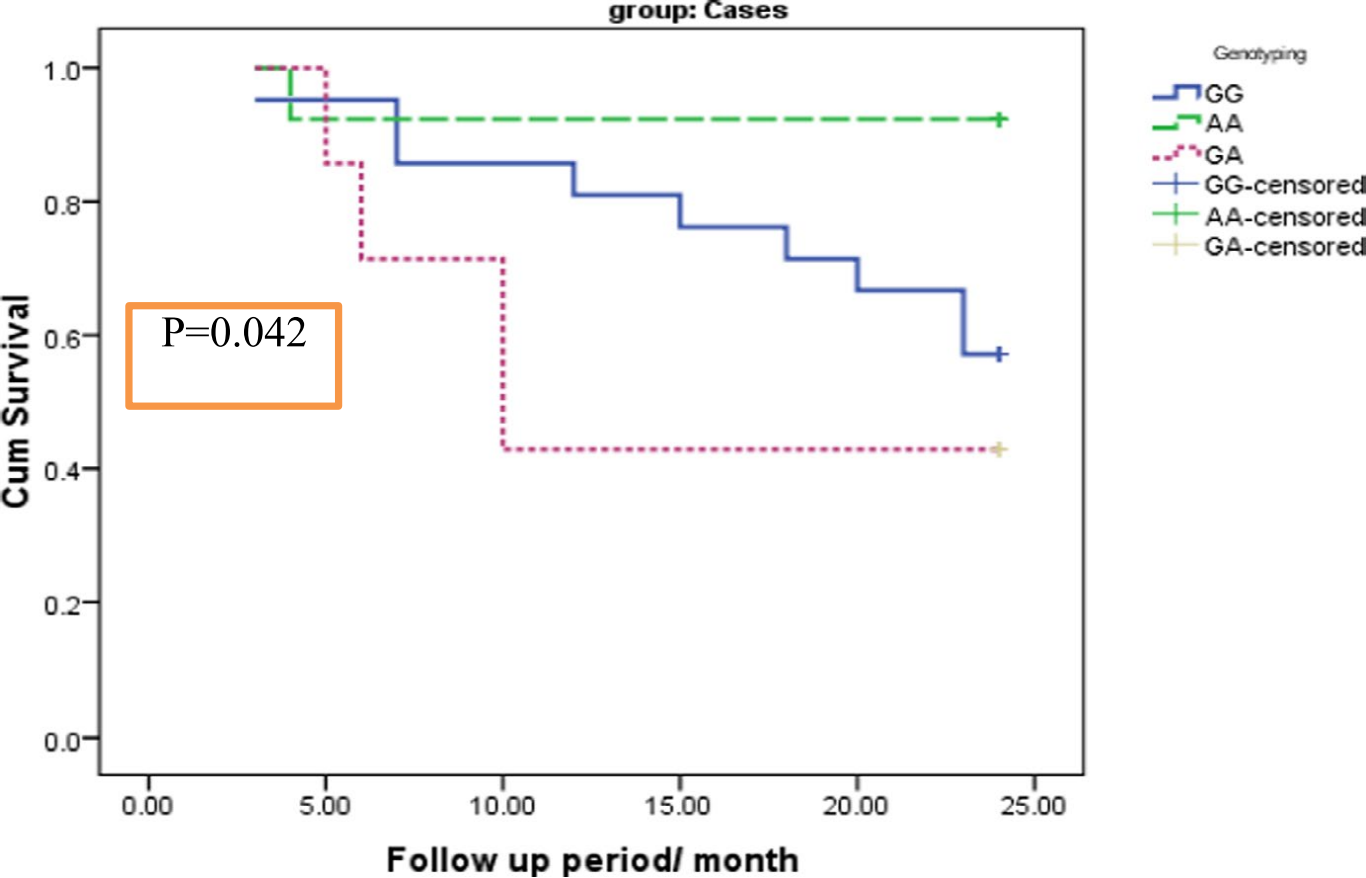
Impact of TERT genotypes on overall survival of MM patients

The impact of TL on the MM patient's OS is presented in Table 7 and Figure 1. MM patients with shorter TL (*P* < .5) had shorter OS as compared to those with long TL (*P* = .049). Moreover, TERT genotype GA had shorter OS as compared to GG and AA genotypes (*P* = .042) (Table 8 and Figure 2).

**Table 7:** Kaplan‐Meier curve for relation between RTL and survival time in case group

RTL	Mean survival time				Log‐rank test	*P*‐value
	Estimate	Std. error	95% confidence interval			
			Lower bound	Upper bound		
RTL <0.5	16.15	2.11	12.00	20.30	7.23	.027*
RTL 0.5‐1	22.20	1.60	19.05	25.34		
RTL >1	21.05	1.54	18.03	24.07		
Overall	19.78	1.10	17.61	21.94		

Abbreviation: RTL, relative telomere length.

* significant

**Table 8:** Kaplan‐Meier curve for relation between genotype and survival time in case group

RTL	Mean survival time				Log‐rank test	*P*‐value
	Estimate	Std. error	95% confidence interval			
			Lower bound	Upper bound		
GG	19.810	1.452	16.964	22.655	6.321	0.042*
AA	22.462	1.478	19.564	25.359		
GA	14.714	3.108	8.622	20.807		

* significant

## 4 DISCUSSION

Cumulative evidence suggests that genetic factors are involved in MM pathogenesis.^19^ Association of telomere length in malignancy is a matter of debate. The genetic variability of the telomerase reverse transcriptase (TERT) could play a role in MM etiology as this gene is responsible for telomere homeostasis and their polymorphic variants have been incriminated in several human cancers.^12^, ^20^, ^21^ Few studies were done concerning the impact of telomere length and genetic polymorphic pattern association as risk factor in those patients.

The main interesting finding in our study was that the telomeres were significantly longer in MM patients as compared to controls in spite the fact of short telomere with age. This finding was in agreement with that reported by Campa et al^22^ who measured the leukocyte telomere length (LTL) in a group of 140 newly diagnosed MM cases and 468 controls; this finding could be explained on the basis of hypothesis by Jones et al,^23^ who concluded that longer telomeres might be a marker of an actively reproducing cell that has an increased chance of acquiring tumor as the telomerase reactivation and telomerase‐mediated elongation of shorter telomeres is a feature of multiple myeloma and TERT inhibition could be serve as a therapeutic strategy approach in future of those MM patients.

Furthermore, explanation for interesting finding, Dagg et al^24^ hypothesized that the initial telomere elongation is caused by a defective trimming of telomeres during embryogenesis, which brings to an unbalanced telomerase activity and then to telomeric over‐lengthening. Similar findings were evident in the study done by Hosnijeh et al_,_ 25 on B‐cell lymphoma patients.

In more addition, Xie et al^26^ observed that relative telomere length was significantly longer in soft‐tissue sarcoma cases than in controls. It has been hypothesized that significantly long telomeres may result in elevated cancer risk by allowing continued cellular proliferation and delaying cellular senescence and apoptosis, thus providing an environment in which cells can accumulate genetic lesions.^27^Rode et al^28^ tested the hypothesis on 95 568 individuals from general population and found that there is an associated long telomeres with increased cancer risk and they concluded that genetic determinants of long telomeres are associated with increased cancer risk, particularly melanoma and lung cancer. This genetic predisposition is going to enhance the telomere maintenance and may represent a survival advantage for pre‐cancerous cells, allowing for multiple cell divisions leading to cancer development.

On the other hand, our observation disagrees with Wu et al^29^ results who revealed significantly reduced telomere length in MM cells compared with telomere length in healthy donor. They also found a strong negative correlation between telomerase activity and telomere length in the MM samples. They explained this finding on the basis of their hypothesis that the telomerase caps the short telomeres, blocking their recognition as damaged DNA.

In the present study among patients' group, there was significant association between stage of the disease and relative telomere length. This finding agrees with Qu et al^30^ who demonstrated that short leukocyte RTL predicts poor prognosis and associated with an immunosuppressive phenotype in the peripheral blood lymphocytes in gastric cancer patients. Also, Chen et al^31^ demonstrated that short leukocyte RTL is an independent bad prognostic marker and associated with the immune functions in colorectal cancer patients.

There was a significant difference between relative telomere length and response of patients to treatment. CR associated with long telomere (RTL > 0.5) however, NR associated with short telomere (RTL < 0.5). This could be suggested that short telomere is associated with more aggressive disease. Similar finding was recently reported by Hyatt et al,^32^ who revealed that patients with short telomeres had a significantly shorter overall survival. Also, in agreement with Jia and Wang^33^; they assessed the prognostic role of telomere length in colorectal cancer that associated with poor overall survival, similar finding was reported by Barczak et al^34^; in breast cancer. In contrast to our finding, Svenson et al^35^ found that patients with long leukocyte telomeres had a significantly worse prostate cancer‐specific and metastasis‐free survival compared to patients with short telomere length. This difference in finding could be explained by different study in different cancer with different tumor biology and different protocol therapy.

In the current study, there was no significant difference between cases and control groups as regard the genotyping analysis of TERT rs2242652. This result is consistent with that reported by Duan et al,^36^ who did not find any significant association of rs2242652 with gastric cancer. Li et al^37^ concluded that there is no correlation between the variant of this SNP and colorectal cancer risk in Chinese Han population. However, it was reported that TERT rs2242652 is associated with significantly higher risks of breast and ovarian cancers^15^ and prostate cancer.^16^

Our finding disagrees with Campa et al^22^ that reported the association of the minor allele of rs2242652 with decreased risk of MM. Zhang et al^38^ found that TERT rs2242652 served as protective factors for the formation of hepatocellular carcinoma, which may be due to the reduced expression of TERT protein by this SNP. These differences in the previous research results may be due to the dysregulated TERT expression in most kinds of tumors. In a study done by Kote–Jarai et al,^39^ they reported that the rs2242652 is associated with decreased TERT expression in prostate cancer and that it is predicted to inhibit the binding of several transcription factors. Bojesen et al^15^ found that the minor alleles of rs2242652 increase silencing and generate a truncated TERT splice variant. These observations would be consistent with a decreased gene expression and activity.

In the present study, there was no significant association of rs2242652 with relative telomere length. This observation was in agreement with Campa et al^22^ However, Bojesen et al^15^ result that this polymorphism was strongly associated with telomere length.

In the present study, there were highly significant differences in age with relative telomere length. The older patients' age was associated with RTL < 0.5 and RTL > 1 was associated with younger patients' age. This result is parallel with previous findings that stated that telomere is shortened with age.^4^, ^40^, ^41^

The limitation of the present study is few patients number. We recommended extension of this study on large cohort group to validate this finding.

## 5 CONCLUSION

Telomere length and TERT genotype (rs2242652) could be used for refining risk stratification of MM patients.

## ETHICAL APPROVAL

This study did not include animals. Informed consent was taken from all subjects who participated in this study.
